# The Role of MCM7 and Its Hosted miR-106b-25 Cluster in Renal Cancer Progression

**DOI:** 10.3390/ijms26178618

**Published:** 2025-09-04

**Authors:** Katarzyna M. Głuchowska, Bartłomiej Hofman

**Affiliations:** 1 Department of Biochemistry and Molecular Biology, Centre of Postgraduate Medical Education, 01-813 Warsaw, Poland; 2 Independent Researcher, 00-735 Warsaw, Poland; hofman.bartlomiej@gmail.com

**Keywords:** renal cancer, *MCM7* gene, miR-106b-25 cluster, ccRCC (clear cell Renal Cell Carcinoma), miR-25, miR-93, miR-106b, *BRMS1L*, *NFIB*, cancer progression

## Abstract

Renal cancer is among the deadliest human malignancies. MCM7, a cell cycle-regulating protein, is frequently overexpressed in cancers and is associated with hyperproliferation and cancer progression. miR-25-3p, miR-93-5p, and miR-106b-5p form the miR-106b-25 cluster, located within the *MCM7* gene, and have previously been reported as upregulated in RCC. This study investigates whether miRNAs from the miR-106b-25 cluster regulate common target genes, enhance one another’s effect, and act synergistically with MCM7 to promote tumor progression. Tissue samples from clear cell RCC (ccRCC) and paired controls were analysed to assess MCM7 expression and genes targeted by the miR-106b-25 cluster. Findings were further validated using the TCGA-KIRC dataset. Functional studies in RCC-derived cell lines were conducted to evaluate the effects of miRNAs on target gene expression, as well as MCM7, and the combined contributions of MCM7 and the miR-106b-25 cluster to renal cancer progression. We demonstrate that MCM7 is upregulated at both transcript and protein levels in RCC, contributing to cancer progression by regulating cell proliferation and caspase-3/7 activity. Furthermore, we identified cancer-related genes aberrantly expressed in ccRCC (*BRMS1L*, *CPEB3*, *DNAJB9*, *KIF3B*, *NFIB*, *PTPRJ*, *RBL2*) and targeted by members of the miR-106b-25 cluster, suggesting that their dysregulation may be driven by these miRNAs. Inhibition of the miR-106b-25 cluster increases caspase-3/7 activity. These findings demonstrate that both MCM7 and the miR-106b-25 cluster contribute to renal cancer progression.

## 1. Introduction

Kidney cancer accounts for approximately 2% of human cancers. In 2022 alone, over 434,000 new cases were diagnosed, and more than 155,000 deaths were attributed to the disease, making it the 16th leading cause of death in adults [1]. Renal Cell Carcinoma (RCC) represents the most common type of kidney cancer, with clear cell Renal Cell Carcinoma (ccRCC, also known as conventional RCC) representing 75–80% of cases [2,3].

ccRCC is characterized by asymptomatic presentation, aggressive growth, and high metastatic potential. In total, 25% of patients present advanced, metastatic disease (mRCC) at first diagnosis, while another 30% develop a spread form of cancer following surgical intervention [2,3].

Although therapeutic strategies for mRCC have expanded in the past decade with the use of targeted therapy, immunotherapy, and personalized treatment, patients’ survival remains poor. Similarly, improvement in understanding the genetic and molecular basis of RCC pathogenesis has revealed different possibilities in the field of biomarkers; none of them have been translated into clinical practice [3,4,5]. More than 50% of mRCC patients die within 2 years of diagnosis, making kidney cancer a significant public health concern and major oncological challenge [2]. Further studies elucidating the mechanism of cancer initiation and progression are critical to establishing not only early detection tools but also more effective targeted therapies—crucial factors for improving patient management and reducing mortality.

MCM7 protein (minichromosome maintenance complex component 7) belongs to the highly conserved MCMs family that forms the MCM2–7 complex. Upregulated in numerous human cancers, it promotes cell proliferation, invasion, and metastasis formation, which was confirmed in vitro and in vivo [6,7]. Elevated MCM7 expression negatively correlates with patient survival in hepatocellular and non-small cell lung cancer [8,9]. Consequently, MCM7 has been described as a tumor-associated gene and is considered a biomarker or even a therapeutic target [10,11,12,13,14].

MCM7 serves as a “host gene” for the miR-106b-25 cluster (formed by three intronic microRNAs (miR-106b-5p, 93-5p, and 25-3p)), which is commonly described as “bi-oncogenic MCM7–miR-106b-25 component” [15].

MicroRNAs (miRNAs, miRs) are short, non-coding ribonucleic acids (RNAs) that primarily function as regulators of target gene expression at the post-transcriptional level. While miRNAs expressed at a physiological level are essential for the development and maintenance of biological processes, their dysregulation has been associated with numerous human cancers. Over the past decades, the field of miRNA cancer research has expanded significantly, advancing understanding of their involvement in tumor biology on the one hand, but also providing their potential for diagnostic and therapeutic application in the clinic on the other [16,17,18]. Similar to MCM7, overexpression of miR-25-3p, miR-93-5p, and miR-106b-5p has been demonstrated in many human cancers, including ccRCC. Their dysregulated levels have been described as one of the factors influencing tumor progression and clinical outcomes, supporting their potential as biomarkers [19,20,21,22].

The involvement of MCM7 in cancer progression and its potential as a diagnostic marker have been proven for human cancers [13,23]. However, MCM7’s role in RCC is rather limited and still needs a deeper understanding. Similarly, the results of elevated levels of miR-25-3p, miR-93-5p, and miR-106b-5p in ccRCC have been characterized for each individually; the whole cluster as an integrated unit has never been investigated in ccRCC. Moreover, the cooperative role of MCM7 and its hosted miR-106b-25 cluster in the progression of ccRCC is still unknown.

In this work, we aimed to explore the consequences of dysregulated levels of MCM7 and the hosted miR-106b-25 cluster on the progression of Renal Cell Carcinoma. We hypothesized that MCM7 levels in ccRCC are dysregulated and impact cancer progression. At the same time, we hypothesized that RNA molecules from the miR-106b-25 cluster commonly target the same dysregulated RCC progression-related genes, cooperate, and, as a unit with MCM7, are involved in ccRCC progression.

## 2. Results

### 2.1. MCM7 Is Significantly Upregulated in ccRCC Patients

**Figure 1:**
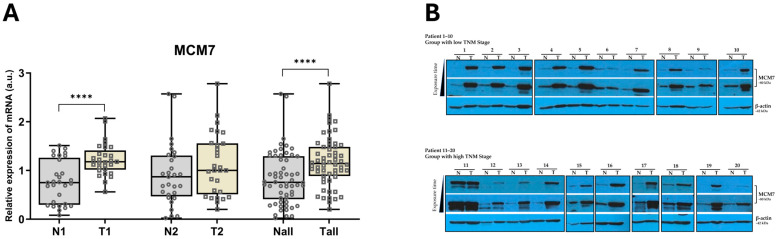
MCM7 is overexpressed in ccRCC tissue samples. (**A**) *MCM7* mRNA expression was analyzed in paired human ccRCC samples from Group 1 (TNM Stages I–II; N1/T1; *n* = 29) and paired samples from Group 2 (TNM Stages III–IV; N2/T2; *n* = 29) using qPCR, with 18S rRNA as the endogenous control gene. Combined analysis of all samples (Nall/Tall, *n* = 58) is also shown. Statistical significance was determined using the Shapiro–Wilk test and the Wilcoxon signed-rank test (**** *p* < 0.0001). (**B**) MCM7 protein level was assessed by Western blot in paired human ccRCC samples. Results from patients P1–P10 (low TNM Stages I–II, *n* = 20) and P11–P20 (high TNM Stages III–IV, *n* = 20) are shown. β-actin was used as the loading control.

First, to elucidate the expression of MCM7 in clear cell Renal Cell Carcinoma (ccRCC), we analyzed RNA and protein samples obtained from human ccRCC specimens. We observed upregulated *MCM7* transcript and protein levels in tumor samples compared to controls (Figure 1). The only group with no significant differences in *MCM7* mRNA levels was Group 2, which comprised TNM Stage III and IV specimens (N2, T2) (Figure 1). To our knowledge, this is the first study to report increased expression of MCM7 protein in patient-derived clear cell Renal Cell Carcinoma (ccRCC) specimens.

**Figure 2:**
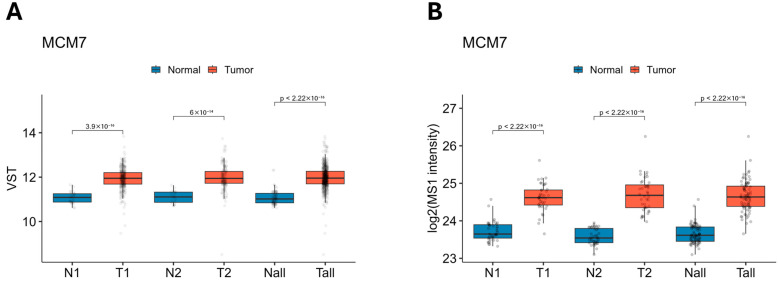
Expression of MCM7 transcript (**A**) and protein (**B**) levels across the TCGA-KIRC and CPTAC-ccRCC datasets. MCM7 expression is elevated in ccRCC at both transcriptomic (**A**) and proteomic (**B**) levels. (**A**) *MCM7* mRNA expression was analyzed using RNA-seq data from TCGA-KIRC. Samples were stratified into low-stage (N1/T1; N1 = 31; T1 = 294; TNM Stage I–II), high-stage (N2/T2; N2 = 27; T2 = 158; TNM Stage III–IV), and all samples combined (Nall/Tall; Nall = 72; Tall = 541). Expression values are shown as variance-stabilized transformed (VST) counts. (**B**) MCM7 protein levels were assessed using CPTAC-ccRCC proteomics data. Log_2_-transformed MS1 intensities are presented for matching sample groupings. Group 1 (Stages I–II; N1/T1; N1 = 40, T1 = 40) and Group 2 (Stages III–IV; N2/T2; N2 = 40, T2 = 40). The combined category included all tumor and normal samples (Nall/Tall; Nall = 80, Tall = 80).

To further validate the observed alterations in MCM7 expression in clinical specimens, we analyzed data from the TCGA-KIRC cohort, which includes a large number of samples. Consistent with our findings, MCM7 was upregulated at both the transcript and protein levels across all analyzed groups, further corroborating our findings (Figure 2).

### 2.2. MCM7 Regulates Properties of Renal Cancer Cells

**Figure 3:**
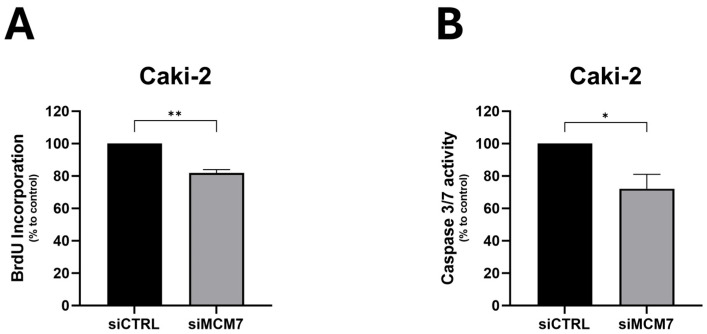
MCM7 inhibition reduces proliferation (**A**) and caspase-3/7 activity (**B**) in renal cancer cells. Caki-2 cells were transfected with synthetic siRNA for MCM7 (siMCM7) or a control oligonucleotide (siCTRL). Proliferation (**A**) and caspase 3/7 activity (**B**) were evaluated 72 h after transient transfection using BrdU and caspase-3/7 activity (**B**) assays, respectively. *n* = 4–5. Results are presented as mean ± SEM. Statistical analysis: Shapiro–Wilk; Mann–Whitney test. * *p* < 0.05, ** *p* < 0.01.

**Figure A1:**
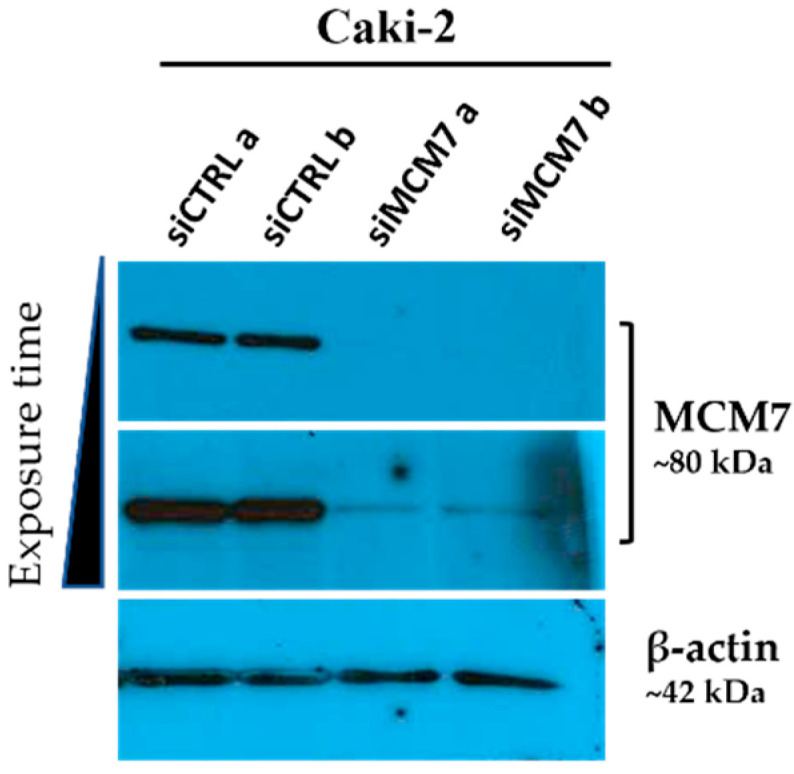
Western blot analysis showing MCM7 gene silencing efficiency. Caki-2 cells were transfected with synthetic siRNA targeting MCM7 (siMCM7) or control oligonucleotides (siCTRL). MCM7 protein levels were evaluated 72 h post-transfection. β-actin was used as a loading control. The results from one biological experiment performed in two replicates are presented.

To study the consequences of upregulated levels of MCM7 in cancer progression, we silenced MCM7 expression in renal cancer cells, Caki-2 (Figure A1), and analyzed the effect on cell behavior. siRNA-mediated MCM7 knockdown significantly reduced proliferation and caspase-3/7 activity of RCC cells (Figure 3). Based on these results, we conclude that MCM7 acts as a tumor-associated gene in kidney cancer.

### 2.3. Targets of miR-106b-25 Cluster Are Abundantly Expressed in ccRCC

Our findings demonstrate that MCM7 is upregulated in clinical samples of ccRCC, and its inhibition reduces cellular proliferation and caspase-3/7 activity in RCC cells, confirming its oncogenic potential. MCM7 forms the MCM7–miR-106b-25 locus together with the miR-106b-25 cluster [15]. miR-25-3p, miR-93-5p, and miR-106b-5p were previously studied in the same RCC specimens analyzed here, and all three, similar to MCM7, were found to be upregulated. Each miRNA, when studied individually, acts as “oncomiR” and triggers cancer progression [20,24]. However, little was known about their cooperative role in the regulation of common target genes in ccRCC, or the function of the MCM7–miR-106b-25 locus as a four-component unit acting simultaneously.

To identify common targets regulated by all three microRNAs from the miR-106b-25 cluster, we employed a comprehensive approach incorporating multiple web-based tools: miRDB, mirDIP, miRSystem, and TargetScan. This analysis identified 92 potential targets (Table A4 in Appendix A). From these, we prioritized genes that: (i) have been implicated in biological processes investigated in this study, (ii) were predicted by at least three algorithms, and (iii) contain miRNA-binding sites within their 3′UTRs as identified by TargetScan. In total, 12 genes–*ATXN1*, *BRMS1L*, *CPEB3*, *COL14A1*, *DNAJB9*, *DOCK4*, *KIF3B*, *NEDD4L*, *NFIB, PTPRJ*, *RBL2*, and *SMAD7*—were selected for further analysis.

**Figure 4:**
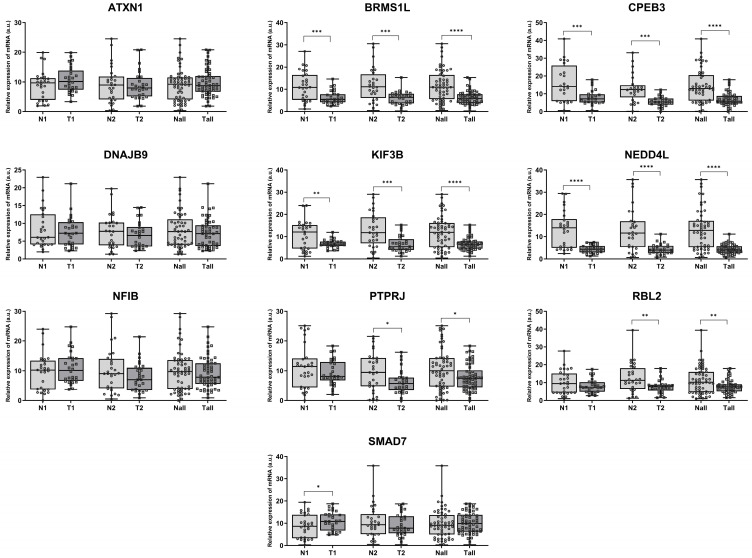
Aberrant expression of cancer progression–related genes in ccRCC. mRNA expression was analyzed in paired human ccRCC samples from Group 1 (TNM Stages I–II; N1/T1; *n* = 29) and paired samples from Group 2 (TNM Stages III–IV; N2/T2; *n* = 29) using qPCR, with 18S rRNA as the endogenous control gene. Combined analysis of all samples (Nall/Tall, *n* = 58) is also shown. Statistical analysis: Shapiro–Wilk test and Wilcoxon signed-rank test (* *p* < 0.05, ** *p* < 0.01, *** *p* < 0.001, **** *p* < 0.0001).

The expression of selected genes was measured by qPCR in the same tissue samples used for MCM7 analysis in this study, as well as previously for members of the miR-106b-25 cluster [20,24]. Considerable changes in expression were observed for 7 out of 12 studied genes. *BRMS1L*, *CPEB3*, *KIF3B*, and *NEDD4L* showed decreased mRNA expression in both low (N1/T1—Group 1) and high (N2/T2—Group 2) stage tumors. *PTPRJ* and *RBL2* were downregulated exclusively in high-stage tumors (N2/T2—Group 2). For all of the aforementioned genes, a significant reduction in mRNA levels was observed when all specimens were analyzed collectively. In contrast, *SMAD7* expression was significantly increased in tissue specimens from patients with TNM Stage I and II (Group 1) (Figure 4). No significant changes were observed for *ATXN1*, *DNAJB9*, and *NFIB*; the expression of *COL14A1* and *DOCK4* was below the detection threshold.

**Figure 5:**
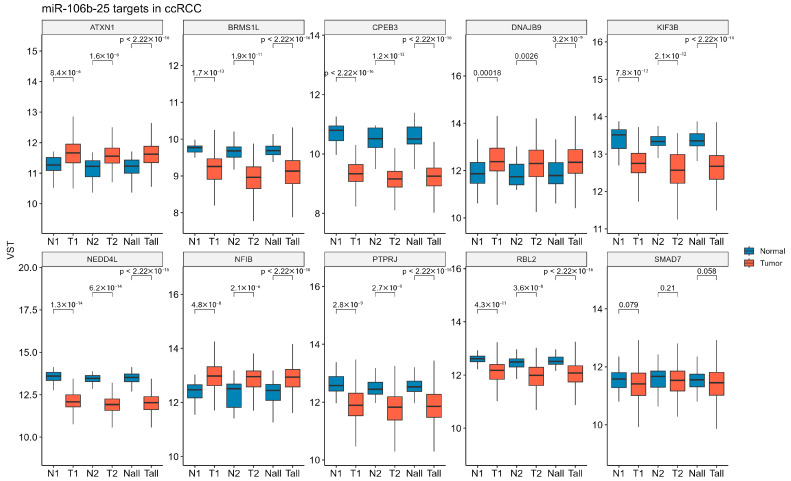
Expression levels of miR-106b-25 targets across the TCGA-KIRC dataset. The mRNA expression levels of 10 predicted miR-106b-25 targets were analyzed using RNA-seq data from TCGA-KIRC. Samples were stratified into low-stage (N1/T1; N1 = 31; T1 = 294; TNM Stage I–II), high-stage (N2/T2; N2 = 27; T2 = 158; TNM Stage III–IV), and all samples combined (Nall/Tall; Nall = 72; Tall = 158). Expression values are shown as variance-stabilized transformed (VST) counts. Statistical differences between groups (N vs. T within each stage) were assessed using the Wilcoxon rank-sum test.

To validate our findings on the expression of ccRCC-progression-related genes in clinical specimens, we analyzed data from a large clinical cohort of KIRC samples in the TCGA database. *ATXN1*, *DNAJB9*, and *NFIB* exhibited increased expression levels in both low- and high-TNM-stage KIRC, as well as in the combined analysis of all samples. *BRMS1L*, *CPEB3*, *KIF3B*, *NEDD4L*, *PTPRJ*, and *RBL2* were significantly downregulated in tumor samples compared to controls. *SMAD7* expression tended to be downregulated across all analyzed sample groups; however, these changes were not statistically significant (Figure 5).

### 2.4. microRNAs from the miR-106b-25 Cluster Regulate the Expression of ccRCC-Progression-Related Genes by Directly Targeting Their 3′UTRs

**Figure 6:**
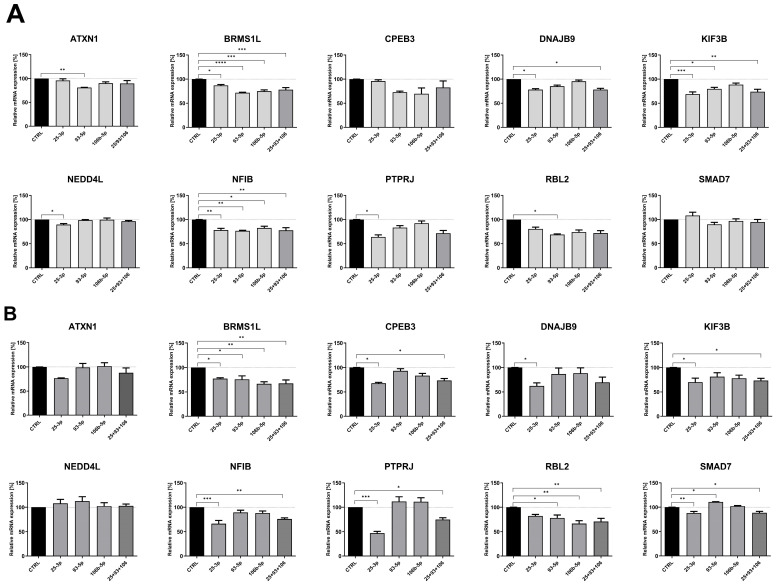
miRNAs from the miR-106b-25 cluster affect the expression of cancer progression-related genes in ccRCC. Caki-2 (**A**) and KIJ-265T (**B**) cells were transfected with synthetic miRNA mimics (for miR-25-35, 93-5p, 106b-5p, or a combination of all three: 25 + 93 + 106) or control oligonucleotides (CTRL). Gene expression was measured by the qPCR method. *n* = 3. Results are presented as mean ± SEM. Statistical analysis: Shapiro–Wilk; ANOVA with Dunn’s post hoc test; Kruskal–Wallis with Dunnett’s post hoc test. * *p* < 0.05, ** *p* < 0.01, *** *p* < 0.001, **** *p* < 0.0001.

To determine if observed changes in identified genes resulted from altered microRNA activity, we transfected two RCC-derived cell lines—Caki-2 and KIJ-265T—with synthetic miRNA mimics. The effect of elevated levels of each miRNA, as well as all three working simultaneously as a “miRNA cluster,” on the studied genes was measured using the qPCR method. In Caki-2 cells, all three microRNAs (miR-25-3p, miR-93-5p, and miR-106b-5p) suppressed expression of *BRMS1L* and *NFIB*. Additionally, miR-25-3p inhibited expression of *DNAJB9*, *KIF3B*, *NEDD4L*, and *PTPRJ*; miR-93-5p downregulated *ATXN1, KIF3B,* and *RBL2*. In a second RCC model—KIJ-265T cells, all three microRNAs negatively regulated *BRMS1L*. Furthermore, miR-25-3p inhibited *CPEB3*, *DNAJB9*, *KIF3B*, *NFIB*, *PTPRJ*, and *SMAD7*; miR-93-5p reduced expression of *RBL2* and slightly enhanced *SMAD7* levels; miR-106b-5p downregulated *RBL2*. Simultaneous co-transfection of cells with all three miRNAs from the miR-106b-25 cluster did not enhance the repressive effect observed with individual miRNAs (Figure 6).

**Figure 7:**
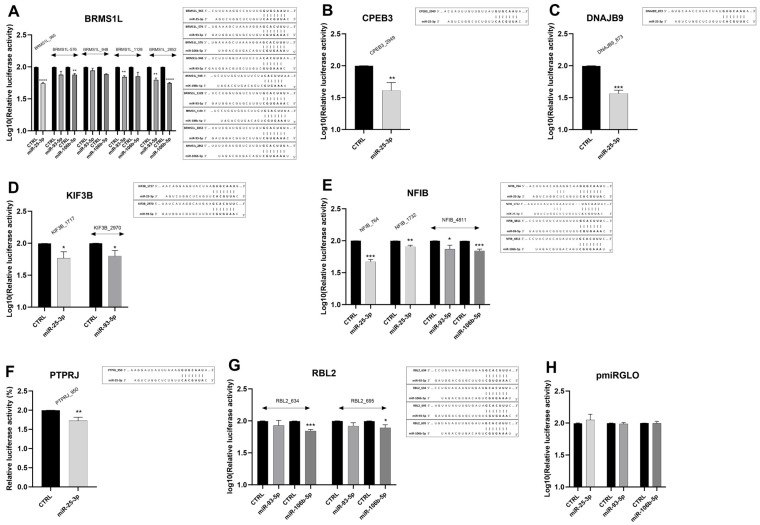
Direct regulation of ccRCC-progression-related genes by miR-25-3p, miR-93-5p, and miR-106b-5p in ccRCC cells. Caki-2 cells were co-transfected with miRNA mimics/control oligonucleotides and constructs (**A**–**G**)/control vector (**H**) as indicated in the Methods section. 48 h after transfection, luciferase activity was measured and normalized to Renilla activity. *n* = 3. Data were log_10_-transformed. Statistical analysis: Shapiro–Wilk, *t*-test, Mann–Whitney test. * *p* < 0.05, ** *p* < 0.01, *** *p* < 0.001, **** *p* < 0.0001. The putative miRNA binding sites in the 3′UTRs of target genes, along with sequence alignments of the miRNA seed regions, are shown to the right of the corresponding graphs.

To further validate the regulatory activity of miRNAs on the studied genes, a luciferase assay was performed. We focused on genes regulated simultaneously by all three microRNAs (*BRMS1L*, *NFIB*), and those exhibiting expression changes exceeding 20% following miRNA overexpression with miRNA mimics (*CPEB3*, *DNAJB9*, *KIF3B*, *PTPRJ*, *RBL2*). Using TargetScan, we identified binding sites in their 3′UTRs with the miRNA-response elements (MREs) and introduced them downstream of the luciferase reporter gene in the pmirGLO vector. Co-transfection of corresponding constructs with miRNA mimics (miR-25-3p, miR-93-5p, or miR-106b-5p) into RCC cells caused a significant inhibition of luciferase activity for all genes chosen for analysis (Figure 7A–G). No changes were observed after co-transfection of mimics with the control pmirGLO plasmid lacking the MREs (Figure 7H).

Collectively, these data indicate that each of the studied genes (*BRMS1L*, *CPEB3*, *DNAJB9*, *KIF3B*, *NFIB*, *PTPRJ*, and *RBL2*) is regulated by at least one of the miRNAs from the miR-106b-25 cluster, and *BRMS1L* and *NFIB* are simultaneously targeted by all three microRNAs. However, no enhanced regulatory effect is observed when all three miRNAs act simultaneously.

### 2.5. Components of the MCM7-miR-106b-25 Locus Affect Properties of RCC Cells

The amplification of the *MCM7–miR-106b-25* locus was reported in human cancers. Studies have shown that each factor derived from the locus exerts mainly oncogenic properties and contributes to cancer progression [6,7,13,21,22,25,26,27,28,29].

However, the expression and role of MCM7 in ccRCC progression remain rather poorly described. The individual roles of miR-25-3p, miR-93-5p, and miR-106b-5p in ccRCC have been previously characterized [20,24,30,31]; the concomitant action of the three microRNAs acting as a single functional factor, as well as the combined role of MCM7 and the miR-106b-25 cluster as a whole, has not been described. Given the fact that all factors from the MCM7-miR-106b-25 locus are overexpressed in ccRCC, we set out to evaluate if they cooperate in processes associated with renal cancer progression.

**Figure A2:**
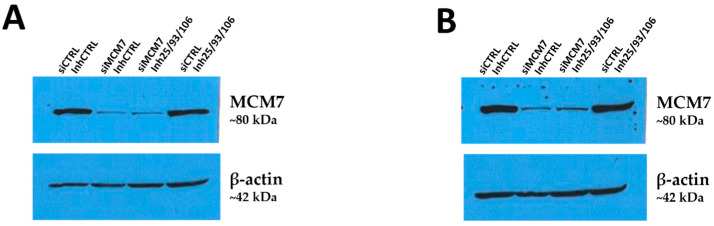
Western blot detection of MCM7 in RCC cells after transfection with siRNA and microRNA inhibitors. Caki-2 (**A**) and KIJ-265T (**B**) cells were co-transfected with siRNA targeting MCM7 (siMCM7) and/or a mix of microRNA inhibitors (Inh25/93/106), as well as corresponding controls (siCTRL, miR-inhibitor control–InhCTRL). MCM7 protein expression was evaluated 72 h post-transfection. β-actin was used as a loading control. Results from one biological experiment are shown.

Two previously adapted cell line models–Caki-2 and KIJ-265T–were co-transfected with siRNA targeting MCM7 and miRNA inhibitors to silence MCM7 alone, miR-106b-25 alone, or both MCM7 and miR-106b-25 simultaneously (Figure A2). The functional effect of these changes on RCC cells’ properties was evaluated using the BrdU assay for cellular proliferation and the caspase-3/7 activity assay.

**Figure 8:**
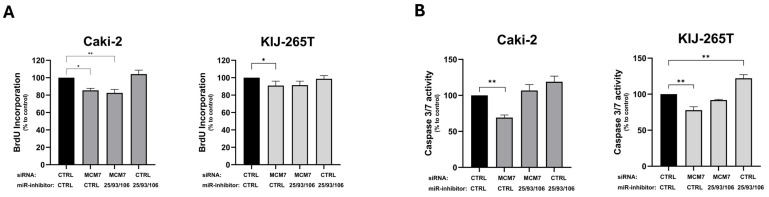
MCM7 and miR-106b-25 cluster alter proliferation (**A**) and caspase-3/7 activity (**B**) of RCC cells. Caki-2 and KIJ-265T cells were co-transfected with siRNA for the MCM7 gene and/or a mix of microRNA inhibitors (miR-inhibitors: 25/93/106) and/or control oligonucleotides (siCTRL, miR-inhibitor CTRL) as shown in the graph description. Cell proliferation (BrdU assay) and caspase-3/7 activity were evaluated 72 h post-transfection. Statistical analysis: ANOVA/Dunn’s test. * *p* < 0.05, ** *p* < 0.01.

Consistent with previous results (Figure 3), reduced MCM7 expression inhibited the proliferation of both Caki-2 and KIJ-265T cells. Although combined inhibition of MCM7 and miR-106b-25 cluster significantly decreased Caki-2 cells’ proliferation, we attribute this effect to MCM7 knockdown, as the inhibition of the miR-106b-25 cluster alone had no impact on proliferation in any RCC cell line used (Figure 8A).

Similarly, reduced MCM7 expression decreased caspase-3/7 activity in both Caki-2 and KIJ-265T cells. In contrast, inhibition of the miR-106b-25 cluster significantly enhanced caspase-3/7 activity in KIJ-265T cells, while a similar trend was observed in Caki-2 cells. Simultaneous reduction in both MCM7 and miR-106b-25 activity showed no effect on caspase-3/7 activity, probably because the individual effects of each factor were abandoned (Figure 8B).

## 3. Discussion

In this study, we demonstrate that MCM7 expression is upregulated in renal cancer and affects cell proliferation and caspase-3/7 activity, contributing to RCC progression. miR-106b-25 cluster contributes to the expression of several key genes, including transcription factors–BRMS1L [32], NFIB [33]; translation and protein stability regulators–CPEB3 [34], DNAJB9 [35]; proto-oncogene–KIF3B [36], and cell signaling molecule–PTPRJ [37]. Most of these genes have been described as tumor suppressors regulating cancer progression. We demonstrate that the expression of these genes is dysregulated in RCC clinical specimens, supporting their potential contribution to the development and progression of RCC. In RCC cells, seven genes (*BRMS1L*, *CPEB3*, *DNAJB9*, *KIF3B*, *NFIB*, *PTPRJ*, *RBL2*) are directly regulated by at least one miRNA from the miR-106b-25 cluster, and two genes–*BRMS1L* and *NFIB*–are simultaneously targeted by all three studied microRNAs. Among the studied microRNAs, miR-25-3p emerges as the most potent regulator, targeting six of the analyzed genes (*BRMS1L*, *CPEB3*, *DNAJB9*, *KIF3B*, *NFIB*, *PTPRJ*). Functional assays demonstrated that MCM7 regulates both proliferation and caspase-3/7 activity in RCC cells, whereas the miR-106b-25 cluster affects caspase-3/7 activity.

MCM7–miR-106b-25 complex consists of MCM7 “host gene” and three intronic microRNAs (miR-106b-5p, 93-5p, and 25-3p), localized on the 7q22 locus frequently amplified in human cancers, including ccRCC [38,39,40]. MCM7 protein is part of the highly conserved MCMs family, which forms the MCM2–7 complex–a key regulator of DNA replication and genome stability. MCM7 is expressed at low levels in normal tissue, but is frequently upregulated in human cancers [28,41,42,43]. High MCM7 expression was correlated with shorter overall survival in several cancers, including hepatocellular carcinoma [8], esophageal squamous cell carcinoma [44], and liver cancer [45], highlighting its potential as a prognostic and predictive biomarker. In pancreatic ductal adenocarcinoma (PDAC) [46], liver [45], breast [47], and colon cancers [46], MCM7 was shown to influence therapeutic response, supporting its promise as a potential therapeutic target.

The role of MCM7 in RCC is not yet fully elucidated. Although *MCM7* expression in ccRCC has been documented using high-throughput approaches [38,48], transcriptional profiling of *MCM7* in ccRCC patients is limited to studies by Liu et al. [49] and Zhang et al. [50], highlighting the need for further validation and assessment at the protein level. Here, we demonstrate that *MCM7* is upregulated at the transcriptional level in ccRCC samples, corroborating previous findings reported by Liu et al. [49] and Zhang et al. [50], and that its protein expression is also elevated in tumor tissue, implicating its role in RCC progression. To our knowledge, this is the first report showing dysregulation of MCM7 at the protein level in ccRCC, as earlier evidence, although consistent with our observations, was limited to immunohistochemical analysis from the Human Protein Atlas [51].

Elevated expression of MCM7 enhances DNA synthesis, promotes cell proliferation, invasion, and contributes to overall cancer progression [7,41,44,45]. To evaluate the functional relevance of the observed MCM7 upregulation in RCC, we knocked down its expression in Caki-2 and KIJ-265T RCC cells, which significantly reduced proliferation and caspase-3/7 activity. These findings align with previous observations in RCC cell lines 786-O and A-498 [50], as well as in hepatocellular carcinoma [6], and esophageal squamous cell carcinoma [44], where MCM7 inhibition similarly suppressed cell proliferation. Most importantly, they confirm the role of MCM7 as a regulator of RCC progression.

Polycistronic microRNAs are often co-dysregulated through shared regulatory mechanisms [52]. Several studies have shown that individual members of the miR-106b-25 cluster are upregulated in ccRCC. When examined separately, each microRNA functions as an “oncomiR”, promoting cancer progression by regulating an individual set of target genes [20,24,30,53,54,55,56,57]. This suggests that in RCC, all three may be dysregulated by a common mechanism and act cooperatively to regulate shared target genes, reinforcing each other’s effect. All three microRNAs were upregulated and repressed NEDD4L [21], EP300 [58], and SMAD7 [59] in breast cancer, influencing epithelial–mesenchymal transition, tumoral transformation, and therapy response. In non-small cell lung cancer, miR-106b-25 cluster targets b-TRCP2 and enhances cell migration and invasion [60], while in acute myeloid leukemia (AML), it targets CASP7, influencing cell proliferation, chemoresistance, and apoptosis [61]. Elevated expression of the entire cluster in chronic lymphocytic leukemia (CLL) [22] and lung cancer [62] further supports their coordinated role.

Using miRNA target prediction tools, we identified 12 genes commonly targeted by all three microRNAs of the miR-106b-25 cluster. Given that MCM7 is primarily involved in cell proliferation–and miRNA from this cluster are also known to regulate this process–we focused on genes related mainly, though not exclusively, to proliferation. The expression levels of *ATXN1*, *BRMS1L*, *COL14A1*, *CPEB3*, *DNAJB9*, *DOCK4*, *KIF3B*, *NEDD4L*, *NFIB*, *PTPRJ*, *RBL2*, *SMAD7* were evaluated in the same ccRCC specimen as MCM7. Among these, six genes–*BRMS1L*, *CPEB3*, *KIF3B*, *NEDD4L*, *PTPRJ*, and *RBL2*–showed significantly decreased expression, while *SMAD7* was significantly upregulated in low-stage tumors. These observations were further supported by KIRC-TCGA data, which confirmed decreased expression of *BRMS1L*, *CPEB3*, *KIF3B*, *NEDD4L*, *PTPRJ*, and *RBL2*.

Given that all three miR-106b-25 members were previously found to be upregulated in the same samples analyzed here [20,24], the observed dysregulation of their potential targets suggests these miRNAs’ role in altered gene expression. In RCC-derived cell lines Caki-2 and KIJ-265T, we demonstrated downregulation of *BRMS1L*, *CPEB3*, *DNAJB9*, *KIF3B*, *NFIB*, *PTPRJ*, and *RBL2* following overexpression of individual microRNA. Specifically, miR-25-3p reduced transcript levels of *BRMS1L*, *CPEB3*, *DNAJB9*, *KIF3B*, *NFIB*, and *PTPRJ*; miR-93-5p suppressed *BRMS1L*, *KIF3B*, and *NFIB*; and miR-106b-5p affected *BRMS1L*, *NFIB*, and *RBL2*. Our studies identify *BRMS1L* and *NFIB* as novel shared targets of all three microRNAs. Among them, miR-25-3p emerges as the most potent regulator, targeting six of the analyzed genes.

Notably, overexpression of each miRNA individually caused reduced expression of *BRMS1L* and *NFIB*; however, simultaneous co-expression of all three miRNAs did not provide an additive or synergistic effect compared to individual miRNAs.

Two–miR-106b-5p and miR-93-5p– microRNAs share the same seed sequence, which supports their ability to regulate common transcripts. miR-25-3p has a distinct seed sequence—implying it targets a different region of the 3′UTR and potentially enhances the miR-93/106b activity [63]. The lack of observable additive or synergistic effects may stem from multiple mechanisms. Beyond target site accessibility and miRNA binding affinity, factors such as mRNA structure and its changes, and competing RNAs can influence miRNA–mRNA interactions and shape the regulatory potential of clustered miRNAs [64,65]. Moreover, a single microRNA can regulate multiple transcripts, while a single transcript may contain binding sites for several microRNAs. This suggests that, at least for miR-93 and miR-106b, concurrent overexpression may lead to competition for binding to shared targets, a phenomenon known as “competitive interaction” [64]. Additionally, miRNAs may also fulfill a redundant role in buffering transcriptomic balance, a function particularly relevant for co-expressed microRNAs that act within the same pathways or biological processes [66].

To fully elucidate the interplay among these miRNAs, further studies addressing the aforementioned mechanisms are required.

BRMS1L (breast cancer metastasis suppressor 1-like) is a transcription factor with tumor-suppressive functions reported in breast [67,68], ovarian [69], and non-small cell lung cancer [32], where it inhibits migration and invasion, thereby limiting metastasis. Its downregulation is associated with metastasis and poor survival in breast [68], ovarian [69], and brain cancers [70], supporting its role as a prognostic biomarker and therapeutic target. To our knowledge, to date, BRMS1L has not been studied in kidney cancer. Based on its function in other malignancies, we hypothesized that BRMS1L may have similar relevance in RCC and serve as a biomarker, and its restoration could offer therapeutic benefits in kidney cancer. This also supports the evidence for the oncogenic potential of miR-106b-25 cluster, which negatively regulates *BRMS1L* expression.

NFIB is a member of the nuclear factor I (NFI) family of transcription factors, with a cancer-specific role [71]. In gastric [72] and colorectal cancer [73], it promotes proliferation, invasion, and metastasis. In contrast, in oral cancer [74] and glioblastoma [75], NFIB acts as a tumor suppressor. In kidney cancer, Wang et al. (2021) [76] reported that NFIB regulates PTEN-induced kinase 1 (PINK1), promoting proliferation, migration, and metastasis formation of RCC. High NFIB expression is associated with poor tumor grade, metastasis, and worse patient prognosis [76]. These findings suggest that NFIB plays an oncogenic role in RCC, and its regulation by the miR-106b-25 cluster, as shown in our study, may have important therapeutic implications.

Studies by Poliseno et al. [15] demonstrated that in prostate cancer, although MCM7 plays a crucial role in tumorigenesis, the simultaneous overexpression and cooperation of the miR-106b-25 cluster is required for oncogenicity. In this study, we show for the first time that the MCM7 protein is upregulated in RCC and confirm its role in cancer proliferation. In contrast to previous reports where individual miRNAs of the cluster promoted renal cancer cell proliferation [20,24,54,77,78,79], concomitant inhibition of the three miRNAs did not change ccRCC proliferation. We observed that MCM7 silencing attenuated the caspase-3/7 activity in both renal cancer cell models–Caki-2 and KIJ-265T. In contrast, simultaneous inhibition of the three miRNAs resulted in upregulation of caspase-3/7 activity. Interestingly, in glioma cells, MCM7 silencing increases caspase 3/7 activity, as reported by Erkan et al. [80]. In alignment with our findings, Hu et al. demonstrated that overexpression of the miR-106b-25 cluster in minimally transformed mammary epithelial cells (MTMECs) suppresses caspase-9 and caspase-3/7 activity [58]. As mentioned previously, in AML, miR-106b-25 targets CASP7 [61]. Further studies are needed to elucidate the functional impact of the altered expression of the MCM7-miR-106b-25 axis in renal cancer.

In conclusion, our study demonstrates that MCM7 mRNA and protein levels are upregulated in renal cancer. The microRNAs–miR-25-3p, 93-5p, and 106b-5p–encoded within the *MCM7* gene–regulate genes that are dysregulated in RCC. Based on our findings, we conclude that elevated MCM7 and miR-106b-25 expression contribute to renal cancer progression.

Despite these benefits, the studies described here have some limitations. As reported, microRNAs operate within complex regulatory networks, often acting redundantly across multiple pathways. Furthermore, cellular pathways are to some degree interrelated, just as one miRNA to another [66]. In our approach, we focus on the regulatory role of a single member or the combined effect of all three members of the miR-106b-25 cluster, with an emphasis specifically on cellular proliferation. While we identified over 90 genes as shared targets of all three miRNAs, each miRNA has hundreds of additional targets not explored here. Likewise, other pathways regulated by the miR-106b-25 cluster may also influence miRNA function and cellular proliferation; however, they were beyond the scope of this study. Further research exploring the interrelations between individual cluster members is needed to comprehensively understand their roles.

Our study provides novel insight into the molecular mechanisms driving RCC progression and highlights potential directions for translational research, which is particularly important given the lack of reliable biomarkers and limited treatment options for advanced RCC [81].

In our study, we observed upregulation of MCM7 protein in RCC tissues compared to matched controls. Taking into account previous reports that identify MCM7 as a sensitive diagnostic marker [10,82], further studies can elucidate its clinical utility in RCC, including its role in shaping therapeutic response [45]. Similarly, the upregulated miRNAs from the miR-106b-25 cluster have previously been reported as potential diagnostic biomarkers, with expression levels correlating with patient outcomes [20,24,79]. In this study, we identified a subset of cancer-related genes regulated by members of this cluster, indicative of a microRNA-mRNA interaction network, and providing novel opportunities for the development of diagnostic biomarkers.

Collectively, our findings broaden knowledge about molecular changes driving RCC progression, supporting its relevance as a candidate for future diagnostic and therapeutic strategies in RCC.

## 4. Materials and Methods

### 4.1. Tissue Specimens

Twenty matched pairs of ccRCC (T) and non-cancerous-adjacent (N) tissue (for protein isolation), along with RNA isolated from 56 matched pairs, were retrieved from The Local Tissue Bank at The Department of Biochemistry and Molecular Biology, Centre of Postgraduate Medical Education. Collection and sample use were authorized by the Institutional Bioethics Committee of the Centre of Postgraduate Medical Education (No. 18/PB/2012, 75/PB-A/2014, 47/PB/2017). Written informed consent was signed by all patients.

### 4.2. Cell Lines Culture

The Caki-2 (HTB-47^™^) RCC-derived cell line was purchased from ATCC (Manassas, VA, USA) and maintained according to the provider’s instructions (McCoy’s 5A Medium Modified (Sigma Aldrich, St. Louis, MO, USA) supplemented with heat-inactivated Fetal Bovine Serum (Sigma Aldrich) to a final concentration of 10% and penicillin/streptomycin (Sigma Aldrich) to a final concentration of 1%). The KIJ-265T cell line was kindly provided by J.A. Copland (Mayo Foundation for Medical Education and Research, Rochester, MN, USA) and maintained in Minimal Essential Medium (MEM, Sigma Aldrich) containing 10% heat-inactivated Fetal Bovin Serum, 1% MEM Non-Essential Amino Acid Solution (MEM NEAA, Sigma Aldrich), 1% sodium pyruvate (Sigma Aldrich) and 1% penicillin/streptomycin (Sigma Aldrich).

### 4.3. Transient Transfections with siRNA and Mimics

All transfections were performed using Lipofectamine^TM^ 2000 reagent (Invitrogen, Carlsbad, CA, USA) according to the manufacturer’s protocol. Cells were seeded in complete medium on 6-well, 12-well, or 96-well plates. The following day, cells were transfected with miRCURY LNA microRNA mimics, inhibitors, or control oligonucleotides (Qiagen, Germantown, MD, USA), anti-MCM7 siRNA, Silencer Select Negative Control No. 1 (Table A1 in Appendix A), or a combination of miRCURY LNA microRNA inhibitors and small interfering RNA (as described in the graphs below). Cultured cells were incubated for 48 h or 72 h and then collected for further post-transfection analysis.

### 4.4. Gene Expression Analysis

RNA/miRNA from cells was isolated using GeneMATRIX Universal RNA/miRNA Purification Kit (EURx, Gdańsk, Poland) according to the provider’s instructions. RNA from matched pairs of ccRCC/control was retrieved from the Tissue Bank.

For real-time quantitative polymerase chain reaction (qPCR) gene expression analysis, cDNA was synthesized using Transcriptor First Strand cDNA Synthesis Kit (Roche Diagnostics, Mannheim, Germany) (for RNA isolated from clinical samples) or RevertAid^TM^ H Minus First Strand cDNA Synthesis Kit (Thermo Fisher Scientific, Rockford, IL, USA) (for RNA isolated from cell cultures) following manufacturer’s instruction. qPCR reactions were performed using SYBR^®^ Green Master Mix (Roche, Mannheim, Germany) and specific primers. Primers sequences used in the study are presented in Table A2 in Appendix A.

For real-time qPCR microRNA expression analysis, cDNA was synthesized using miRCURY LNA^TM^ Universal cDNA Synthesis Kit (Qiagen, Germantown, MD, USA) according to the provider’s protocol. qPCR reactions were performed using ExiLENT SYBR^®^ Green Master Mix (Qiagen, Germantown, MD, USA) and miRCURY LNA^TM^ miRNA PCR primers (Qiagen, Germantown, MD, USA). Primers sequences used in the study are presented in Table A3 in Appendix A.

### 4.5. Total Protein Isolation and Western Blotting Analysis

Tumor tissues and cultured cells were homogenized in RIPA buffer (Thermo Fisher) supplemented with protease inhibitors (Roche) and β-mercaptoethanol. Protein extracts (30 µg from tissue or 25 µg from culture cells) were separated on a 12% SDS-PAGE gel and transferred to a nitrocellulose membrane (Thermo Fisher). Membranes were blocked in 5% non-fat milk (in TBST) and incubated with primary antibodies: mouse-anti-MCM7 (Novus Biologicals, #H00004176-M01; 1:500, Centennial, CO, USA), mouse-anti-β-actin (Abcam, #ab6276; 1:10,000, Cambridge, UK), followed by secondary antibody incubation (anti-mouse goat, Dako; 1:10,000, Santa Clara, CA, USA). Signals were visualized using ECL substrate (SuperSignal^TM^ West Pico PLUS, ThermoFisher) and developed on X-ray film (Kodak, Rochester, NY, USA). β-actin was used as a loading control.

### 4.6. Functional Assays

#### 4.6.1. Luciferase Reporter Assay

Luciferase activity was determined 48 h after transfection using the Dual-Glo Luciferase Reporter Assay System (Promega, Madison, WI, USA) as described [20]. Briefly, Caki-2 cells were co-transfected with constructs (pmirGLO Dual-Luciferase miRNA Target Expression Vector (Promega)) containing miRNAs binding sites in the 3′UTR of target genes or control vector and miRCURY LNA microRNA mimics or negative control (Qiagen, Germantown, MD, USA). Luciferase activity was normalized to Renilla and presented as relative luciferase activity.

#### 4.6.2. BrdU Incorporation Assay

Cell proliferation was measured 72 h after transfection using the BrdU incorporation assay according to the manufacturer’s instructions.

#### 4.6.3. Caspase-3/7 Activity Assay

Caspase-3/7 activity was measured 72 h after transfection using the Caspase-Glo^®^ 3/7 Assay Kit (Promega, Madison, WI, USA) following the manufacturer’s instructions.

### 4.7. Bioinformatics, TCGA, and CPTAC Data Analysis

To identify the putative miRNA target genes, we used several prediction tools, including miRDB [83,84], mirDIP [85,86], miRSystem [87], and TargetScan [88] to visualize the miRNA binding sites in the 3′UTR of selected target genes.

To verify our findings in the larger ccRCC cohort, we utilized datasets from The Cancer Genome Atlas (TCGA) and the Clinical Proteomic Tumor Analysis Consortium (CPTAC).

RNA-seq data for KIRC cancer were obtained from TCGA via the TCGAbiolinks package [89]. For the analysis, samples labelled as “Primary Tumor” and “Solid Tissue Normal” were selected. The final dataset consisted of 541 tumor samples and 72 normal samples. Raw count data from the TCGA-KIRC cohort were obtained using the TCGAbiolinks package and processed in R (v4.5.1) [90]. Genes were filtered to retain only entries annotated as *protein_coding*, based on Ensembl metadata. One sample labeled as “Additional—New Primary” (barcode: TCGA-DV-A4W0-05A-11R-A266-07) was excluded due to inconsistent classification. A variance stabilizing transformation (VST) was applied to raw counts using the vst function from the DESeq2 package (v1.40.2) [91], yielding normalized expression values suitable for visualization and hypothesis testing. Samples were stratified according to TNM classification into two clinical groups: Group 1 (Stages I–II; N1/T1; N1 = 31, T1 = 294) and Group 2 (Stages III–IV; N2/T2; N2 = 27, T2 = 158). An additional combined category included all tumor and normal samples (Nall/Tall; Nall = 72, Tall = 541). mRNA expression comparisons between tumor and normal samples were performed using the Wilcoxon rank-sum test, and corresponding *p*-values were used to assess statistical significance. Selected genes of interest were visualized using boxplots and statistical annotations generated with ggplot2 [92].

Proteomics data for clear cell Renal Cell Carcinoma (CCRCC) were obtained from the LinkedOmics database [93], specifically from the CPTAC CCRCC cohort included in the CPTAC pan-cancer initiative [94]. The dataset included quantitative protein expression profiles derived from mass spectrometry-based proteomics and consisted of 103 tumor samples and 80 normal samples. Only samples with MCM7 quantified were selected for further analysis (80 tumor samples and 80 normal samples). Samples were labelled according to TNM classification into two clinical groups: Group 1 (Stages I-II; N1/T1; N1 = 40, T1 = 40) and Group 2 (Stages III-IV; N2/T2; N2 = 40, T2 = 40). An additional combined category included all tumor and normal samples (Nall/Tall; Nall = 80, Tall = 80). Protein abundance values from paired tumors and adjacent normal tissues were merged and filtered to retain proteins with non-missing values across all samples. Patients were stratified based on TNM stage classification (Stage I–II vs. Stage III–IV), allowing stage-specific comparisons. Protein expression levels between tumor and normal samples were compared using the Wilcoxon rank-sum test, applied independently for each protein.

### 4.8. Statistical Analysis and Data Presentation

All experiments were conducted in at least three independent biological repetitions, each performed in triplicate, unless otherwise indicated in the figure legends. GraphPad Prism 10 (GraphPad Software Inc., Boston, MA, USA) was used for statistical analysis. Data distribution was examined by the Shapiro–Wilk test. Differences between groups were measured by *t*-test, Kruskal–Wallis test, or Analysis of Variance (ANOVA), followed by appropriate post-hoc comparison. For the analysis of clinical samples, the Wilcoxon signed-rank test was used.

## 5. Conclusions

Taken together, our findings support an important role for MCM7 and the miR-106b-25 cluster in renal cancer. MCM7 is upregulated in renal cancer at the mRNA and protein levels, and its dysregulation contributes to cell proliferation and caspase-3/7 activity. MicroRNAs from the intragenic miR-106b-25 cluster regulate the expression of cancer-related genes found to be dysregulated in renal cancer and modulate caspase-3/7 activity. These findings underscore the oncogenic potential of the MCM7 and miR-106b-25 cluster, supporting its relevance as a candidate for future diagnostic and therapeutic strategies in RCC.

## Data Availability

Data is contained within the article: the original contributions presented in this study are included in the article. Further inquiries can be directed to the corresponding author.

## Appendix A

**Table A1:** List of miRCURY LNA ^TM^ miRNA mimic/inhibitor/control nucleotides used in the study (purchased from Qiagen (Hilden, Germany)).

Product Name/Target Name	Cat. Number
hsa-miR-25-3p miRCURY LNA miRNA Mimic	339173_YM00470873-ADA
hsa-miR-93-5p miRCURY LNA miRNA Mimic	339173_YM00471046-ADA
hsa-miR-106b-5p miRCURY LNA miRNA Mimic	339173_YM00471973-ADA
Negative Control miRCURY LNA miRNA Mimic	339173_YM00479902-ADA
hsa-miR-25-3p miRCURY LNA miRNA Inhibitor	339121_YI04100613-ADA
hsa-miR-93-5p miRCURY LNA miRNA Inhibitor	339121_YI04101031-ADA
hsa-miR-106b-5p miRCURY LNA miRNA Inhibitor	339121_YI04100930-ADA
Negative Control miRCURY LNA miRNA Inhibitor	339126_YI00199006-ADA

**Table A2:** List of primers used for real-time qPCR analysis.

Gene Name Abbreviation	Nucleotide Sequence (5′ → 3′)	Source
18sRNA	F: GTAACCCGTTGAACCCCATT R: CCATCCAATCGGTAGTAGCG	[95]
ACTB	F: CCAGCTCACCATGGATGATG R: ATGCCGGAGCCGTTGTC	[96]
ACTB	F: CGGCATCGTCACCAACTG R: GCTGGGGTGTTGAAGGTCTC	[97]
ATXN1	F: ACAGTGGAACCTATGCCAGC R: GTTGGCCAGCAGAGTGGAAT	This study
BRMS1L	F: GGCACAGCATTGATATTACCTCA R: TATGGACCTGAAACAACAACTGG	[69]
COL19A1	F: GTGACCCGATTGCACTTCC R: TGGCCCTATATCACCCTTCCG	This study
CPEB3	F: CCCTTCTCCAGCAACGTGAT R: TCATAGGGCCTACTCCGGTC	This study
DOCK4	F: TCCTACTTTTACCTCGCAGTC R: TCCCAGCTTCCTTGTTATGGAT	This study
DNAJB9	F: CCAAAATCGGCATCAGAGCG R: ATTTTGCTTCAGCATCCGGG	This study
KIF3B	F: CTACCAACATGAACGAGCAC R: GGTTCAATTTTCCTACACGGAT	This study
MCM7	F: CCCCTCCCAGTTTGAACCTCT R: TCGCCTCATCTCCACGTATGCT	This study
NEDD4L	F: TGCCCAAACCACCCCGTCAT R: TCAGGACTGCCCCATTGCTC	[98]
NFIB95	F: GCCCATAACCCAGGGAACTG R: CCTCTGAAGATTGACCCCCG	This study
PTPRJ	F: CTGTTTCCATCAGTCCAACC R: ATGAAGTCGCTGGACGTAAG	[99]
RBL2	F: CAACAATGGGCAAACGGTAAC R: GCCACTTGACCAGGGACTTG	[100]
SMAD7	F: ATGATCTACCTCAGGGGAAT R: GACTTGATGAAGATGGGGTA	[101]

**Table A3:** List of miRCURY LNA ^TM^ miRNA PCR Assays (LNA primers) used in the study (purchased from Qiagen (Hilden, Germany)).

Product Name/Target Name	Cat. Number
hsa-miR-25-3p miRCURY LNA miRNA PCR Assay	339306_YP00204361
hsa-miR-93-5p miRCURY LNA miRNA PCR Assay	339306_YP00204715
hsa-miR-106b-5p miRCURY LNA miRNA PCR Assay	339306_YP00205884
SNORD44(hsa) miRCURY LNA miRNA PCR Assay	339306_YP00203902
hsa-miR-28-5p miRCURY LNA miRNA PCR Assay	339306_YP00204322
hsa-miR-103a-3p miRCURY LNA miRNA PCR Assay	339306_YP00204063

**Table A4:** List of genes predicted as common targets for all three microRNAs (miR-25-3p, miR-93-5p, miR-106b-5p).

ABCG4 AFF1 AFF4 ANKIB1 ANKRD13C ATXN1 B3GALT2 BCL11B BCL2L11 BMPR2 BRMS1L CD69 C17ORF39 CHRM2 CHD9 CIC COL19A1 CPEB3 DLGAP2 DNAJB9 DNAJC27 DOCK4 E2F3 EGR2 FAM126B FAM117B FAM19A1 FCHO2 FNDC3B FRS2 FXR1 GATAD2B HEG1 GIT2 GNS GOLGA1 GPR137C IKZF4 KIAA1128 KIF3B LUZP1 JOSD1 KAT2B LYST MCL1 MTF1 MYT1L NEDD4L NFAT5 NFIB NR4A3 ODZ1 OTUD4 PAFAH1B1 PALLD PHTF2 PITPNA PIP5K3 PTEN PTPRD PTPRJ PTPRO RAB8B RBL2 RGL1 RNF38 RSBN1 RGMA SDC2 SERTAD2 SH3PXD2A SIRPA SMAD6 SMAD7 SGK269 SNF1LK SNIP SOBP SOCS6 SOX4 TTC9 UBE2W USP28 WASL WDFY3 XRN1 TWF1 UBASH3B UBE2W USP28 XRN1 ZNF148
